# Impact of Dolutegravir-Based Antiretroviral Therapy on Piperaquine Exposure following Dihydroartemisinin-Piperaquine Intermittent Preventive Treatment of Malaria in Pregnant Women Living with HIV

**DOI:** 10.1128/aac.00584-22

**Published:** 2022-11-14

**Authors:** Clifford G. Banda, Dumisile Nkosi, Elizabeth Allen, Lesley Workman, Mwayiwawo Madanitsa, Marumbo Chirwa, Mayamiko Kapulula, Sharon Muyaya, Steven Munharo, Joel Tarning, Kamija S. Phiri, Victor Mwapasa, Feiko O. ter Kuile, Gary Maartens, Karen I. Barnes

**Affiliations:** a Malawi-Liverpool-Wellcome Research Programme, Blantyre, Malawi; b Division of Clinical Pharmacology, Department of Medicine, University of Cape Town, Cape Town, South Africa; c Kamuzu University of Health Sciences (formerly College of Medicine and Kamuzu College of Nursing, University of Malawi), Blantyre, Malawi; d WorldWide Antimalarial Resistance Network (WWARN), Pharmacology Scientific Group, University of Cape Town, Cape Town, South Africa; e Training and Research Unit of Excellence, Blantyre, Malawi; f Department of Clinical Sciences, Malawi University of Science and Technology, Limbe, Malawi; g Centre for Tropical Medicine and Global Health, Nuffield Department of Medicine, University of Oxford, Oxford, United Kingdom; h Mahidol-Oxford Tropical Medicine Research Unit, Faculty of Tropical Medicine, Mahidol University, Bangkok, Thailand; i Department of Clinical Sciences, Liverpool School of Tropical Medicine, Liverpool, United Kingdom

**Keywords:** piperaquine, dihydroartemisinin-piperaquine, antiretroviral therapy, HIV, malaria, pregnancy, intermittent preventive treatment

## Abstract

Dihydroartemisinin-piperaquine, an artemisinin-based combination therapy, has been identified as a promising agent for intermittent preventive treatment of malaria in pregnancy. However, in pregnant women living with HIV (PLWH), efavirenz-based antiretroviral therapy (ART) significantly reduces the plasma exposure of piperaquine. In an open-label, nonrandomized, fixed-sequence, pharmacokinetic study, we compared piperaquine plasma concentrations in 13 pregnant women during a 3-day treatment course of dihydroartemisinin-piperaquine when coadministered with efavirenz-based versus dolutegravir-based ART in the second or third trimester of pregnancy. Piperaquine concentrations were measured over a 28-day period, while on efavirenz-based ART and after switching to dolutegravir-based ART. Noncompartmental analysis was performed, and geometric mean ratios (GMRs) and 90% confidence intervals (CIs) were generated to compare piperaquine pharmacokinetic parameters between these two treatment periods. Compared with efavirenz-based ART, coadministration of dihydroartemisinin-piperaquine and dolutegravir-based ART resulted in a 57% higher overall piperaquine exposure (area under the concentration-time curve from 0 to 672 h [AUC_0–672 h_]) (GMR, 1.57; 90% CI, 1.28 to 1.93). Piperaquine’s day 7 concentrations were also 63% higher (GMR, 1.63; 90% CI, 1.29 to 2.11), while day 28 concentrations were nearly three times higher (GMR, 2.96; 90% CI, 2.25 to 4.07). However, the maximum piperaquine concentration (*C*_max_) remained similar (GMR, 1.09; 90% CI, 0.79 to 1.49). Dihydroartemisinin-piperaquine was well tolerated, with no medication-related serious adverse events observed in this small study. Compared with efavirenz-based ART, a known inducer of piperaquine metabolism, dolutegravir-based ART resulted in increased overall piperaquine exposure with pharmacokinetic parameter values that were similar to those published previously for pregnant and nonpregnant women. Our findings suggest that the efficacy of dihydroartemisinin-piperaquine will be retained in pregnant women on dolutegravir. (The study was registered on PACTR.samrc.ac.za [PACTR201910580840196].)

## INTRODUCTION

In most areas of malaria endemicity in sub-Saharan Africa, HIV is also highly prevalent (1). In these settings, pregnant women are at increased risk of malaria-associated adverse outcomes, including maternal anemia, severe malaria, low birthweight, and stillbirth deliveries. The risk is higher in pregnant women living with HIV (PLWH) (2–6), necessitating prioritizing malaria control in this high-risk subpopulation.

One of the tools for malaria prevention is to use intermittent preventive treatment during the second and third trimesters of pregnancy (IPTp) in moderate- to high-intensity malaria transmission settings. This strategy involves regular administration of standard treatment doses of antimalarial drugs during pregnancy. In pregnant women who are HIV negative, the antimalarial drug currently recommended by WHO is sulfadoxine-pyrimethamine (SP). However, the efficacy of SP is being undermined by increasing resistance of malaria parasites (7–9). PLWH cannot receive SP for IPTp if they are already on another sulfonamide-based combination with daily trimethoprim-sulfamethoxazole (co-trimoxazole) to prevent HIV-associated opportunistic infections. Intermittent administration of dihydroartemisinin-piperaquine has thus been suggested as an effective alternative to SP in pregnant women (7, 8, 10) and is being explored as add-on therapy to daily co-trimoxazole in PLWH and on antiretroviral therapy (ART) (11, 12).

Dihydroartemisinin-piperaquine is an artemisinin-based combination therapy (ACT) that contains a short-acting artemisinin derivative, dihydroartemisinin, and a longer-acting partner drug, piperaquine. During IPTp, dihydroartemisinin rapidly suppresses any parasite load while piperaquine clears any remaining parasites and confers protection against new infections due to its long half-life (13). In a previous study in Uganda, efavirenz-based ART led to a 38% reduction in piperaquine exposure in PLWH compared with HIV-negative pregnant women (14). This reduction has the potential to lower the therapeutic efficacy of dihydroartemisinin-piperaquine. The mechanism of efavirenz’s effect on piperaquine’s exposure is likely due to induction of CYP3A4, which metabolizes piperaquine (15, 16). This has led to several modeling exercises to define dose adjustments of dihydroartemisinin-piperaquine needed for IPTp when coadministered with efavirenz-based ART (17, 18).

As countries in sub-Saharan Africa increase the rollout of the integrase strand transfer inhibitor dolutegravir to replace efavirenz, we aimed to assess whether dolutegravir-based ART, compared with efavirenz-based ART, is associated with adequate piperaquine pharmacokinetic exposure when administered as a 3-day IPTp course of dihydroartemisinin-piperaquine in a cohort of pregnant women in Malawi.

## RESULTS

### Study profile.

**FIG 1:**
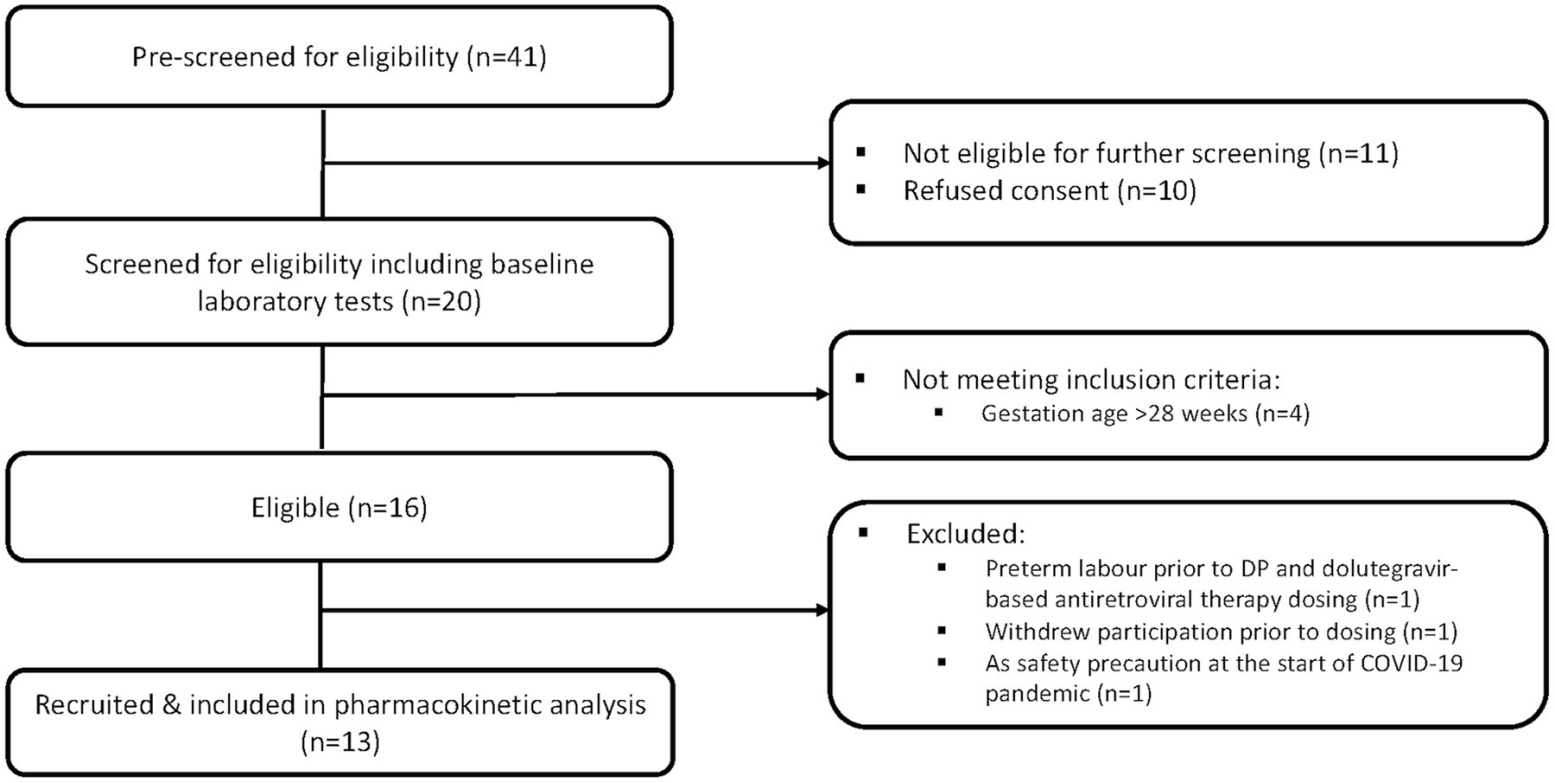
Participant flow chart.

Twenty pregnant women were screened for eligibility, and 13 were recruited into the study (Fig. 1). The baseline characteristics of the 13 participants are summarized in Table 1. Nearly a third of the participants (4/13) were receiving tuberculosis prophylaxis with isoniazid and pyridoxine, initiated when they started ART in line with the national HIV treatment policy at that time.

**TABLE 1:** Baseline characteristics of study participants included in the analysis

Characteristic	Value (*n* = 13)
Age in yr (median [range])	35 [20–42]
Ultrasound-estimated gestational age in wk (median [range])	21 [17–27]
Second trimester (*n* [%])	12 [92.3]
Third trimester (*n* [%])	1 [7.7]
Wt (kg, median [range])	63.3 [47.2–77.8]
Total administered dose of piperaquine (mg/kg of body wt, median [range])	
Sequence 1	55.3 [49.6–62.6]
Sequence 3	55.3 [48.2–61.7]
BMI^*a*^ (kg/m^2^, median [range])	24. 7 [19.6–32.1]
CD4 count (cells/mm^3^, median [range])	589 [339–1,219]
Hemoglobin (g/dL, median [range])	10.9 [8.8–13.7]
Concomitant medication (*n* [%])	
Co-trimoxazole prophylaxis	13 [100]
Isoniazid prophylaxis and pyridoxine	4 [30.8]
Ferrous sulfate plus folic acid	13 [100]

a BMI, body mass index.

### Pharmacokinetics of piperaquine.

**FIG 2:**
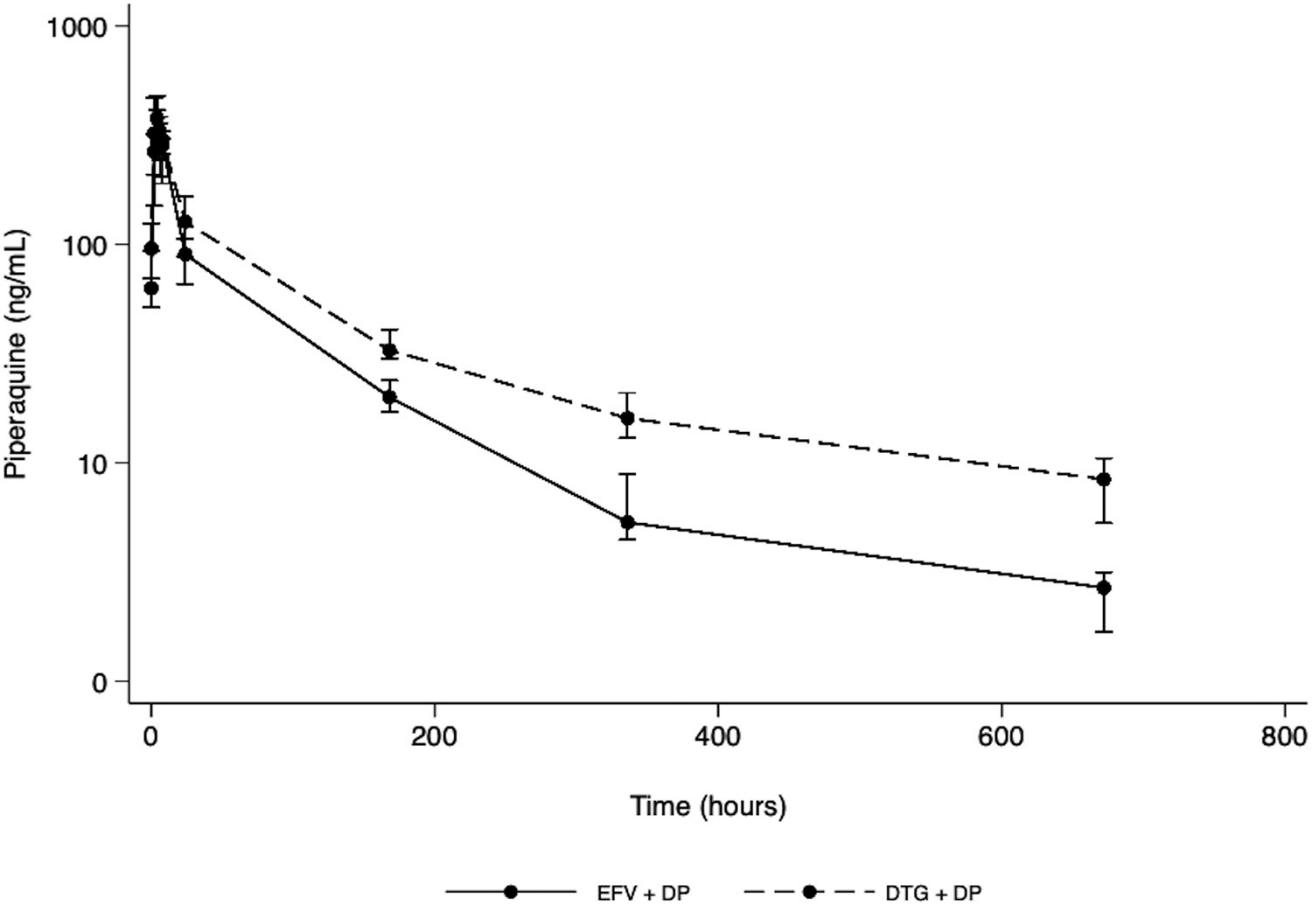
Plasma piperaquine concentration-time profile following coadministration of dihydroartemisinin-piperaquine with efavirenz (EFV)-based antiretroviral therapy (solid line) and with dolutegravir (DTG)-based antiretroviral therapy (dashed line) in 13 pregnant women. Data are presented on a semilogarithmic plot as medians and interquartile ranges.

The pharmacokinetic parameters of piperaquine were compared while participants were on efavirenz-based ART (sequence 1) and again 6 weeks later after they had received dolutegravir-based ART for 1 month (sequence 3) following a switch in ART regimens as per national guidelines. Sequence 1 data spanned from 0 to 14 days and included a day 28 (672-h) piperaquine sample collected in sequence 2 once the participant had switched to dolutegravir-based ART. Efavirenz has been previously reported to remain detectable several weeks after stopping therapy (19) and has an autoinduction effect on CYP3A4 enzymes that metabolize piperaquine, which was expected to wane gradually (20). Therefore, despite participants being on dolutegravir, this day 28 sample was aimed at capturing piperaquine trough concentrations in the terminal elimination curve of efavirenz following the switch from efavirenz to dolutegravir. For purposes of this study, sequence 1 data referred to piperaquine concentrations from 0 to 14 days as well as day 28 (672 h). Sequence 3 piperaquine data encompassed concentrations from 0 to 672 h (day 28). A separate comparison of dolutegravir pharmacokinetic profile when administered without and with dihydroartemisinin-piperaquine in sequences 2 and 3, respectively, has been reported previously (21). Piperaquine pharmacokinetic parameters and concentration profiles, upon completion of a 3-day treatment course of dihydroartemisinin-piperaquine in sequences 1 and 3, are shown in Table 2 and Fig. 2, respectively. Compared with efavirenz, coadministration of dihydroartemisinin-piperaquine and dolutegravir resulted in a 57% higher (geometric mean ratio [GMR], 1.57; 90% confidence interval [CI], 1.28 to 1.93) overall piperaquine exposure (area under the concentration-time curve from 0 to 672 h [AUC_0–672 h_]). The maximum piperaquine concentration (*C*_max_) remained similar (GMR, 1.09; 90% CI, 0.79 to 1.49).

**TABLE 2:** Piperaquine exposure during coadministration with dolutegravir-based antiretroviral therapy compared with efavirenz-based antiretroviral therapy^*a*^

Pharmacokinetic parameter	GM (90% CI)		GM ratio (90% CI) for sequence 3/sequence 1	*P* value^*b*^
	Piperaquine on DTG-based ART (sequence 3)	Piperaquine on EFV-based ART (sequence 1)		
AUC_0–672 h_ (ng · h/mL)	25,904 (22,322–30,061)	16,492 (14,317–18,998)	**1.57 (1.28–1.93)**	**0.001**
*C*_max_ (ng/mL)	424 (339–532)	390 (314–484)	1.09 (0.79–1.49)	0.511
*C*_day 7_ (ng/mL)	31 (27–37)	19 (16–23)	**1.63 (1.29–2.11)**	**<0.001**
*C*_day 28_ (ng/mL)	8.0 (6.5–10.0)	2.7 (2.2–3.2)	**2.96 (2.25–4.07)**	**<0.001**
*T*_max_ (h)	3.5 (2.9–4.3)	3.8 (3.0–4.9)	0.92 (0.67–1.27)	0.682
*t*_1/2_ (h)	260 (219–309)	190 (169–215)	**1.37 (1.10–1.69)**	**0.014**
CL/F (L/h)	138 (119–160)	213 (188–241)	**0.65 (0.53–0.79)**	**0.001**

a GM, geometric mean; DTG, dolutegravir; EFV, efavirenz; ART, antiretroviral therapy; CI, confidence interval. Bold represents statistical significance.

b Paired *t* test.

Furthermore, piperaquine’s day 7 concentrations (*C*_day 7_) were 63% higher (GMR, 1.63; 90% CI, 1.29 to 2.11), while day 28 concentrations (*C*_day 28_) were nearly three times higher (GMR, 2.96; 90% CI, 2.25 to 4.07). The proportion of participants who achieved a piperaquine *C*_day 28_ of 10 ng/mL, previously predicted to provide a 95% probability of being parasitemia free during pregnancy (22), was 30% and 0% while on dolutegravir and efavirenz, respectively. These proportions were 76% and 8%, respectively, when a lower protective threshold of 5 ng/mL was considered.

### Impact of concomitant isoniazid prophylaxis.

Since isoniazid is known to inhibit CYP3A4 (23), a main metabolic pathway for piperaquine, *post hoc* analyses were stratified by concomitant isoniazid use to explore any potential impact of isoniazid on plasma piperaquine exposure. Among the nine participants who were not on isoniazid prophylaxis, the overall piperaquine exposure was 64% higher (GMR, 1.64; 90% CI, 1.26 to 2.12) when on dolutegravir-based ART compared with efavirenz-based ART (see Table S1 and Fig. S1 in the supplemental material). In the four participants who were on isoniazid prophylaxis, there was a modest increase in piperaquine exposure of 43% (GMR, 1.43; 90% CI, 0.94 to 2.17) when on dolutegravir-based ART compared with efavirenz-based antiretroviral therapy (Table S1).

### Effect of trimester on piperaquine concentration.

Of the 12 participants who were in the second trimester of pregnancy at enrollment in sequence 1 (Table 1), 6 had progressed to the third trimester by the time sequence 3 was conducted, while the remaining 6 were still in the second trimester. To account for the potential effect of trimester change, and other independent variables, on piperaquine pharmacokinetic exposure parameters (AUC_0–672 h_, *C*_max_, *C*_day 7_, and C_day 28_), a mixed-effect regression analysis was conducted. The regression analysis showed that a change in trimester did not independently affect piperaquine pharmacokinetic parameters (Table S2).

### Treatment-emergent adverse events.

Table 3 summarizes treatment-emergent adverse events stratified by the period when they occurred. There were 82 reported adverse events throughout the study period, only one of which was considered related to the administration of dihydroartemisinin-piperaquine (Table S3). This was a mild case of vomiting, which occurred 30 min after administering the first dose of dihydroartemisinin-piperaquine. There were no further episodes of vomiting in this participant after the second and third doses. In sequence 1, while participants were on efavirenz-based ART, all 32 events (100%) were mild. In sequence 3, while participants were on dolutegravir-based ART, there were 27 adverse events, all of which were mild except for one moderate adverse event of catheter site pain requiring paracetamol analgesia.

**TABLE 3:** Treatment-emergent adverse events grouped by period of occurrence and severity

Adverse event classified according to MedDRA system	Sequence 1^*b*^				Intersection between sequences 1 and 2^*c*^				Sequence 3^*d*^				Delivery^*e*^				Total
	Mild	Moderate	Severe	Life threatening	Mild	Moderate	Severe	Life threatening	Mild	Moderate	Severe	Life threatening	Mild	Moderate	Severe	Life threatening	
Cardiac disorders																	
Palpitations	1								1								2
Orthostatic hypotension									1								1
Gastrointestinal disorders																	
Abdominal discomfort					1												1
Abdominal pain	1																1
Diarrhea	2																2
Nausea	6					1			3								10
Toothache					2				1								3
Vomiting	7				2				4								13
Upper gastrointestinal hemorrhage^*a*^						1											1
General disorders and administration site conditions: catheter site pain	2									1							3
Immune system disorders: hypersensitivity									1								1
Infections and infestations																	
Gastroenteritis					1												1
Rhinitis	1				1												2
Trichomoniasis					1												1
Upper respiratory tract infection	1								2								3
Urinary tract infection	1				3	1			2								7
Musculoskeletal disorders																	
Arthralgia	1								1								2
Musculoskeletal pain	1								1								2
Nervous system disorders																	
Dizziness	5								2								7
Headache					2				2								4
Hypoesthesia	1				1				1								3
Pregnancy, puerperium, and perinatal conditions																	
Neonatal asphyxia^*a*^																1	1
Premature rupture of membranes^*a*^															1		1
Preterm labor^*a*^													1				1
Reproductive system and breast disorders: vaginal discharge	1																1
Skin and subcutaneous tissue disorders																	
Night sweats									1								1
Pruritus					1	0											1
Pruritic rash	1				1	1			3								6

Total	32				16	4			26	1			1		1	1	82

a Serious adverse event.

b Two-week period following coadministration of efavirenz-based ART and dihydroartemisinin-piperaquine.

c Four-week lead-in period while on dolutegravir-based ART following a switch from efavirenz-based ART; period also reflecting elimination phase of piperaquine initial dihydroartemisinin-piperaquine dose.

d Within the first 28 days of dihydroartemisinin-piperaquine and dolutegravir-based ART coadministration plus any events presenting as unscheduled visits prior to delivery.

e Occurring at delivery, >4 weeks after last dose of dihydroartemisinin-piperaquine, while on dolutegravir-based ART.

There were four serious adverse events that led to hospitalization or prolonged hospitalization (Table 3). The first event was for upper gastrointestinal intestinal bleeding presenting as hematemesis, considered probably due to a Mallory-Weiss tear. This occurred at 26 weeks of gestation and was of moderate severity. The participant required hospitalization for observation, and no other subsequent events occurred. The second event was a urinary tract infection-associated premature rupture of membranes at 33 weeks of gestation that progressed to preterm delivery, as a third event, and resulted in the birth of a male infant weighing 2.6 kg. The infant was admitted to the hospital for a 3-day observation period per local guidelines and recovered. The last event was an early neonatal death due to birth asphyxia that resulted from a prolonged second stage of labor during a term delivery. This led to prolonged hospitalization of the baby, who died on day 2 of admission to the pediatric intensive care unit. None of these serious adverse events were assessed as related to the coadministration of dihydroartemisinin-piperaquine and antiretroviral therapy (Table S3).

### Pregnancy outcomes and viral load changes during follow up.

There was one early neonatal death due to birth asphyxia, as noted above. The median birthweight in the 12 live infants was 3.0 (range, 1.5 to 3.8) kg. Viral load remained suppressed (below 50 copies/mL) in all 13 enrolled participants throughout the study.

## DISCUSSION

We investigated the impact of dolutegravir-based ART, compared with efavirenz-based ART, on the pharmacokinetic profile of piperaquine in pregnant women living with HIV in the second and third trimesters of pregnancy. Unlike efavirenz-based ART, dolutegravir-based ART resulted in increased overall piperaquine exposure with pharmacokinetic parameter values similar to those previously published for pregnant and nonpregnant women. Furthermore, there were no differences in the safety profile of dihydroartemisinin-piperaquine in the two treatment periods. Our findings are reassuring as they suggest that dolutegravir, in contrast to efavirenz, can be administered with a standard treatment course of dihydroartemisinin-piperaquine in pregnancy without increasing the risk of adverse effects or reducing piperaquine exposure (14).

The mechanism behind the observed higher piperaquine exposure (57% higher AUC_0–672 h_) when participants were on dolutegravir than when they were on efavirenz is likely due to the lack of an induction effect by dolutegravir on CYP3A4, which metabolizes piperaquine (15). While efavirenz is a known inducer of this family of enzymes (16) and results in reduced piperaquine exposure, dolutegravir is not an inducer or inhibitor of CYP3A4 (24, 25). Additionally, dihydroartemisinin is metabolized by UDP-glucuronosyltransferase (UGT1A9 and UGT2B7) (26). Thus, dolutegravir is not expected to impact the metabolism of dihydroartemisinin-piperaquine.

The concentrations of piperaquine, when administered with dolutegravir, fall within ranges previously described in pharmacokinetic studies of piperaquine in pregnant and nonpregnant women for malaria prevention and treatment (14, 27, 28) (see Table S4 in the supplemental material). In these studies, the observed exposure of piperaquine was well tolerated and was not associated with cardiac toxicity, which is concentration dependent (29). Notably, in our present study, there was no significant difference in the maximum concentration (*C*_max_) between the two treatment periods (Table 2). Additionally, there were no differences in the occurrence and severity of adverse events when dihydroartemisinin-piperaquine was coadministered with either of the two antiretroviral therapy regimens (Table 3), with only one episode of postdose vomiting considered to be associated with dihydroartemisinin-piperaquine administration but not associated with higher piperaquine exposure. Moreover, unlike with efavirenz-based ART, dolutegravir-based ART was not accompanied by lower piperaquine concentrations on day 7 (*C*_day 7_), which correlate with overall exposure, are predictive of malaria treatment success (30), and could help ensure dihydroartemisinin-piperaquine’s effectiveness in treating any preexisting malaria parasitemia during pregnancy.

However, although coadministration of dihydroartemisinin-piperaquine with dolutegravir resulted in higher day 28 piperaquine concentrations (Table 2), the proportion of participants achieving a day 28 concentration of 10 ng/mL, which has been predicted to provide a 95% probability of being parasitemia free during pregnancy (17, 22), was low (30% while on dolutegravir and 0% while on efavirenz). This could suggest that monthly dosing of dihydroartemisinin-piperaquine with dolutegravir or efavirenz may not provide adequate minimum protective exposure. Since the 10-ng/mL threshold was first predicted from studies conducted in HIV-negative women, it may not be an accurate protective threshold in women living with HIV who concurrently receive co-trimoxazole prophylaxis given that there could be a potential additive malaria-protective effect from the co-trimoxazole. Thus, when a lower *C*_day 28_ protective threshold of 5 ng/mL was explored in this study, as has been previously suggested (17), there was an improvement in the proportion of participants who achieved piperaquine concentrations that were above this lower protective threshold (76% while on dolutegravir and 8% while on efavirenz). Future studies should aim to quantify the additive protective effect of co-trimoxazole during malaria preventive treatment with dihydroartemisinin-piperaquine in pregnant women living with HIV.

In our cohort, progression from second to third trimesters of pregnancy did not seem to impact piperaquine exposure (Table S2). However, only six women had their dihydroartemisinin-piperaquine dose in this period; therefore, these findings need to be interpreted with caution. Nevertheless, they indicate that dihydroartemisinin-piperaquine can be administered in the second or third trimester of pregnancy without concern for a significant variation in drug exposure. This observation is also consistent with previously described findings of a lack of effect of gestational age on piperaquine exposure in HIV-negative pregnant women (31).

The combination of dolutegravir-based ART and dihydroartemisinin-piperaquine was well tolerated in this small study. These safety profiles are consistent with previously approved drug labels for both treatments (32, 33). There were no changes in viral load following the antimalarial-antiretroviral treatment combination.

**FIG 3:**
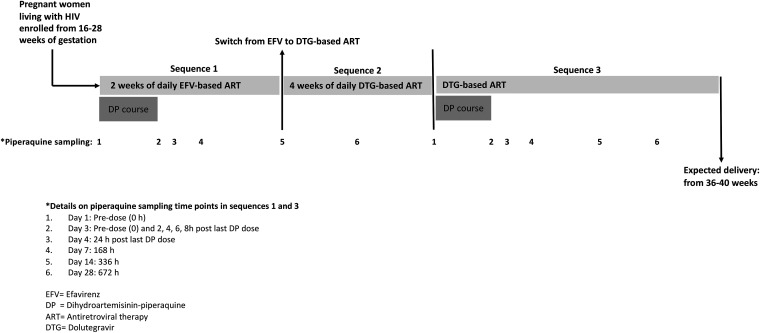
Study design. A fixed-sequence study switching from efavirenz- to dolutegravir-based antiretroviral therapy. Intense plasma pharmacokinetic sampling of piperaquine was conducted in two sequences: when participants were on efavirenz-based antiretroviral therapy (sequence 1) and when they were on dolutegravir-based antiretroviral therapy (sequence 3) following a 4-week lead-in period from the time of switching from efavirenz- to dolutegravir-based antiretroviral therapy.

Our study has limitations. First, due to the fixed sequence design, we were unable to precisely differentiate the impact of antiretroviral therapy on piperaquine exposure from any period effect or sequence effect. However, the present design captured the period effect or sequence effect by increasing gestational age. Since we did not observe an impact of increasing gestation age (data not shown), or progression from second to third trimester (Table S2), on piperaquine exposure, the findings in our study can be attributed reasonably to the effect of antiretroviral therapy on piperaquine exposure. Second, the national policy to switch pregnant women from efavirenz-based ART to dolutegravir-based ART resulted in administering efavirenz for 14 and not 28 days (Fig. 3). This may have underestimated the impact of efavirenz on piperaquine exposure, especially when AUC was calculated from 0 to 672 h (day 28). However, an exploratory sensitivity analysis showed that when AUC calculation was based on data from 0 to 336 h in both treatment periods, dolutegravir-based ART, compared with efavirenz-based ART, still resulted in increased piperaquine exposure with pharmacokinetic parameter values (Table S5) that were similar to those published previously for pregnant and nonpregnant women. Third, only a small number of women on isoniazid prophylaxis were included in this study, given the changes in national policy for tuberculosis preventive treatment with isoniazid. We were, therefore, unable to accurately assess the impact of isoniazid on piperaquine exposure when coadministered with dihydroartemisinin-piperaquine (Fig. S1 and Table S1); adequately powered, prospective pharmacokinetic studies are needed to investigate this question. Fourth, due to safety restrictions put in place to mitigate the spread of SARS-CoV-2, we were unable to recruit the planned 16 participants. However, the recruited 13 participants in our cohort provided adequate power (80%) to detect a change in AUC outside the FDA limits for bioequivalence for piperaquine when comparing piperaquine’s AUCs while participants were on efavirenz- and dolutegravir-based ART (34).

In conclusion, compared with efavirenz-based ART, a known inducer of piperaquine metabolism, dolutegravir-based ART resulted in increased piperaquine exposure when administered as a standard treatment course of dihydroartemisinin-piperaquine, with pharmacokinetic parameter values and safety profiles that were similar to those published previously for pregnant and nonpregnant women. This ensures dihydroartemisinin-piperaquine’s predicted treatment and protective efficacy in this vulnerable subpopulation without any clinically significant adverse events observed in this small study.

## MATERIALS AND METHODS

### Study design and patient selection.

We conducted an open-label, nonrandomized, fixed-sequence, pharmacokinetic study between December 2019 and July 2020 in PLWH at a clinical research facility within a tertiary hospital, in Zomba district, in the southern region of Malawi. The study protocol was approved by the Malawian College of Medicine Research Ethics Committee (P.07/19/2746), the University of Cape Town’s Human Research Ethics Committee (266/2019), and the Liverpool School of Tropical Medicine’s Research Ethics Committee (19-039). The study was registered on PACTR.samrc.ac.za (PACTR201910580840196).

Prior to screening for eligibility, potential participants were identified and prescreened at the antenatal and ART clinics. Prescreening involved ascertaining, from the hospital records, the HIV status and ART regimen being received as well as estimating gestational age from the date of last menstrual period before ultrasound scanning. Potential participants who were willing to further discuss the study information were taken through the consenting process before commencing with screening procedures.

The inclusion criteria were as follows: (i) adult pregnant women (≥18 years of age) with no symptoms of malaria, presenting at the hospital for antenatal care from 16 to 28 weeks of gestation (confirmed by ultrasound scan); (ii) virologically suppressed (viral load of <50 copies/mL) on an efavirenz-based antiretroviral regimen; (iii) CD4 cell count of >100 cells/mm^3^; (iv) resident within the hospital catchment area; and (v) willing to adhere to follow-up procedures including intensive pharmacokinetic sampling. Exclusion criteria were as follows: (i) multiple pregnancies; (ii) severe malformations or nonviable pregnancy found by ultrasound scan; (iii) known allergy or contraindication to any study drug; (iv) use of medications known or suspected to interact with dolutegravir or piperaquine other than those recommended by national treatment guidelines, such as isoniazid; and (v) medical history of comorbidities likely to influence pharmacokinetic parameters of study drugs, such as renal, liver, or cardiac disease.

A sample size of 14 participants was calculated to have at least 80% power to detect a change in AUC outside the FDA limits for bioequivalence for piperaquine (34). After accounting for a 10% loss to follow-up, 16 participants were planned for recruitment.

### Study procedures and pharmacokinetic blood sampling.

Figure 3 summarizes the study sequences, dosing schedule, and piperaquine pharmacokinetic blood sampling time points. Participants who provided written informed consent received an IPTp course comprising fixed-dose tablets of dihydroartemisinin-piperaquine (D’Artepp, Guilin, China) given once a day for three consecutive days in sequence 1, while on daily efavirenz-based ART, and again in sequence 3, while on dolutegravir-based ART. Each fixed-dose combination tablet contained 40 mg of dihydroartemisinin and 320 mg of piperaquine. Participants were dosed according to body weight and in line with the manufacturer’s specifications, as follows: three, four, or five tablets if weighing 36 to 59 kg, 60 to 79 kg, and ≥80 kg, respectively. Efavirenz was administered as a fixed daily combination dose of 300 mg tenofovir-300 mg lamivudine-600 mg efavirenz (Symfi; Mylan, USA). Daily dolutegravir-based antiretroviral therapy (Reydin film-coated tablets; Cipla, India) contained a fixed dose of 50 mg of dolutegravir, 300 mg of lamivudine, and 300 mg of tenofovir disoproxil fumarate.

In sequence 1, participants received a 3-day IPTp course of dihydroartemisinin-piperaquine, with the first and third doses being observed. On the last dosing day (day 3 of dihydroartemisinin-piperaquine treatment), intensive blood sampling for piperaquine plasma concentrations was conducted predose (0 h) and at 2, 4, 6, 8, and 24 h after observed dosing of dihydroartemisinin-piperaquine and efavirenz-based ART. Thereafter, samples were collected on days 7 (168 h), 14 (336 h), and 28 (672 h). However, due to a national policy change that switched pregnant women from efavirenz- to dolutegravir-based ART, efavirenz was continued only to day 14 and not day 28. The switch from efavirenz- to dolutegravir-based ART on day 14 commenced sequence 2 (Fig. 3). Nevertheless, a piperaquine sample was collected on day 28 (672 h) when the participant was 2 weeks into sequence 2. The day 28 sampling was aimed at capturing piperaquine trough concentrations in the terminal elimination phase of efavirenz following the switch to dolutegravir. This was done since efavirenz has been previously reported to remain detectable several weeks after stopping therapy (19) and has a gradually reducing autoinduction effect on CYP3A4 enzymes that metabolize piperaquine (20).

In sequence 2, participants were on dolutegravir-based ART for 4 weeks as shown in Fig. 3. The 4-week lead-in period allowed dolutegravir concentrations to attain steady state and allowed for the waning of efavirenz induction on enzymes (CYP3A4) that metabolize piperaquine. In sequence 3, participants received a 3-day IPTp course of dihydroartemisinin-piperaquine, with first and last doses again being observed and intensive pharmacokinetic sampling for piperaquine conducted at the same time points as for sequence 1 (Fig. 3).

Since efavirenz was usually administered in the evening, participants were instructed to take permitted concomitant medications (e.g., isoniazid and co-trimoxazole as well as a fixed-dose combination of ferrous sulfate and folic acid) in the morning. The reverse was advised when the women were switched to dolutegravir-based antiretroviral therapy to avoid the effects of concomitant iron on dolutegravir absorption (35). To standardize timing between food and antiretroviral therapy or dihydroartemisinin-piperaquine intake, participants were instructed to take medications at least 2 h before or after meals. An electronic device (Wisepill RT 2000; Wisepill Technologies, Somerset West, South Africa) was used to monitor daily drug intake for dihydroartemisinin-piperaquine and antiretroviral therapy. Participants were reminded, through short text messages, if a dose was missed. Routine antenatal care continued in parallel to all the described study procedures.

### Safety assessments.

At screening, a detailed medical history, a physical examination, and an ultrasound scan were performed. Thereafter, participants were followed up until delivery. At each follow-up visit, symptom-directed history taking and physical examination were conducted. Adverse events and details on prescription medicines, herbal supplements, over-the-counter medications, dietary supplements (vitamins included), or vaccines since the last visit were elicited using open questions. All adverse events detected at scheduled or unscheduled visits were recorded, graded, and independently assessed by two physician investigators to classify any possible, probable, or definite relationships to dihydroartemisinin-piperaquine and antiretroviral therapy coadministration. Prior to dihydroartemisinin-piperaquine dosing, HIV viral load was measured. This was repeated just before sequence 3 and 28 days after coadministration of dihydroartemisinin-piperaquine with dolutegravir to assess whether participants remained virologically suppressed.

### Piperaquine blood sampling and quantification.

Piperaquine blood samples were collected in EDTA-coated tubes. Within 5 min of collection, samples were centrifuged and separated into cryovials containing 200 μL of plasma. The plasma samples were temporarily stored at −20°C before being transferred, within a week of collection, for storage at −80°C until shipment to the Mahidol-Oxford Tropical Medicine Research Unit (MORU) in Thailand. Piperaquine plasma concentrations were measured using solid-phase extraction followed by liquid chromatography coupled with tandem mass spectrometry according to a previously reported method (36). Quality control (QC) samples at 4.50, 20.0, and 400 ng/mL were analyzed in triplicate within each batch of clinical samples to ensure the accuracy and precision of the assay. The percent relative standard deviations (%RSDs) at low, middle, and high concentration levels were 4.70%, 4.38%, and 4.92%, respectively. The limit of detection (LOD) and the lower limit of quantification (LLOQ) were set to 0.375 and 1.50 ng/mL, respectively. The laboratory at MORU participates in the quality assurance/quality control (QA/QC) proficiency testing program supported by the WorldWide Antimalarial Resistance Network (WWARN) (37).

### Statistical analysis.

Data from sequences 1 (piperaquine concentrations while on efavirenz-based ART) and 3 (piperaquine concentrations while on dolutegravir-based ART) were analyzed. Using noncompartmental analysis, employing the trapezoidal rule with cubic splines, the following pharmacokinetic parameters were estimated for the two treatment periods: the area under the concentration-time curve to the last measurable time point at 672 h (28 days) postdosing (AUC_0–672 h_), terminal elimination half-life (*t*_1/2_), maximum concentration (*C*_max_), and time to *C*_max_ (*T*_max_). The apparent clearance (CL/F) of piperaquine was calculated using the equation dose/AUC_0–672 h_, while the concentrations known to correlate with overall exposure and dihydroartemisinin-piperaquine’s malaria treatment efficacy were estimated from the sample collected on day 7 (*C*_day 7_). Furthermore, the minimum protective concentration of piperaquine, following the monthly treatment course, was estimated from samples collected on day 28 (*C*_day 28_). The proportion of participants achieving a day 28 concentration of ≥10 ng/mL, which has been previously predicted to provide a 95% probability of being parasitemia free during pregnancy (17, 22), was calculated in each dosing period. Additionally, a lower protective threshold of 5 ng/mL was explored, as previously suggested for pregnant women living with HIV and on co-trimoxazole prophylaxis (17).

Pharmacokinetic data were log transformed to calculate the geometric mean ratio (GMR) of sequence 3 to sequence 1 pharmacokinetic parameters with their 90% confidence intervals (CIs). Subsequently, the log-transformed pharmacokinetic parameters were compared between the two sequences using paired *t* tests and back-transformed to absolute nanogram-per-milliliter concentrations for reporting. Changes in pharmacokinetic parameters between the two sequences were considered statistically significant when the 90% CI of the GMR did not cross the value of 1 (34). These analyses were further stratified by concomitant isoniazid use *post hoc*, to explore the impact of isoniazid prophylaxis on the plasma exposure of piperaquine when coadministered with efavirenz- or dolutegravir-based ART; isoniazid is known to inhibit CYP3A4 enzymes (23), a main metabolic pathway for piperaquine. All analyses were performed using Stata version 15.1.
