# Supplementary material for: Impact of Dolutegravir-Based Antiretroviral Therapy on Piperaquine Exposure following Dihydroartemisinin-Piperaquine Intermittent Preventive Treatment of Malaria in Pregnant Women Living with HIV
Source: Antimicrob Agents Chemother. 2022 Nov 14;66(12):e00584-22. doi: 10.1128/aac.00584-22 (PMC9764988; doi:10.1128/aac.00584-22)
Supplement: Supplemental file 1 — Supplemental material. Download aac.00584-22-s0001.pdf, PDF file, 0.3 MB [file aac.00584-22-s0001.pdf]

SUPPLEMENTARY MATERIAL LEGENDS

Figure S1. Plasma piperaquine concentration-time profile following coadministration of dihydroartemisinin-piperaquine with efavirenz (EFV)-based antiretroviral therapy (solid line) and with dolutegravir (DTG)-based antiretroviral (dashed line) in 9 pregnant women who were not on daily isoniazid prophylaxis (graph plots on the left), and in 4 pregnant women who were on isoniazid prophylaxis (graph plots on the right). Data are presented on a semi-logarithmic plot as medians (interquartile ranges [IQR]).

Table S1. Piperaquine exposure when administered with dolutegravir- compared with efavirenz-based antiretroviral therapy, stratified by concomitant intake of isoniazid prophylaxis.

Table S2. Mixed-effect regression analysis assessing the impact of independent variables on piperaquine exposure.

Table S3. Relationship between treatment-emergent adverse events and coadministration of dihydroartemisinin with antiretroviral therapy, stratified by severity and period of follow-up.

Table S4. Overview of published data on the pharmacokinetics of piperaquine in pregnancy, postpartum, and non-pregnant women.

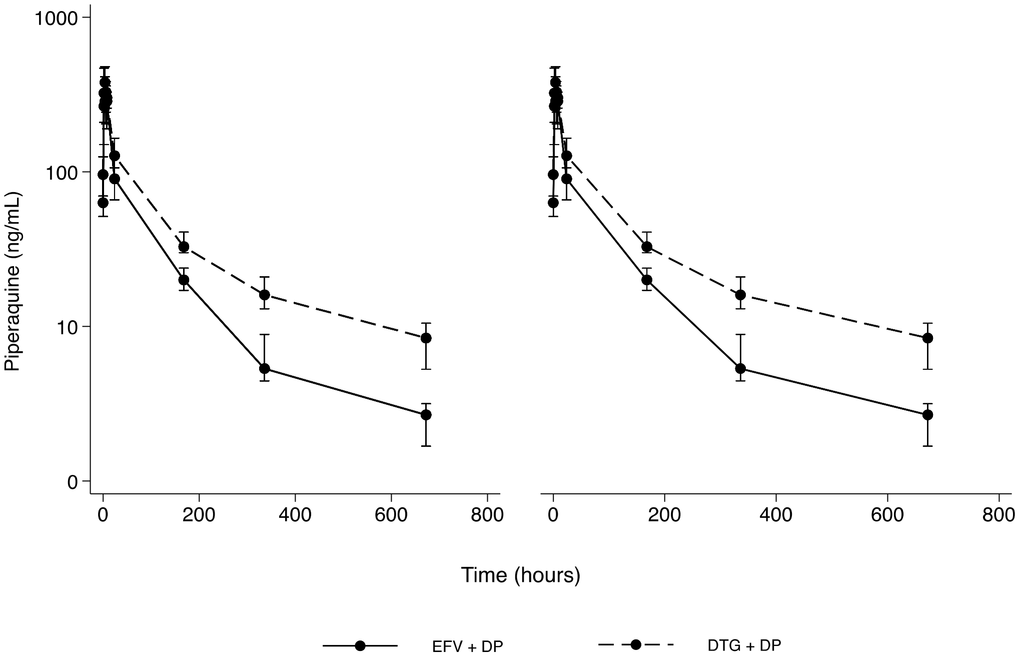

Figure S1. Plasma piperaquine concentration-time profile following coadministration of dihydroartemisinin-piperaquine with efavirenz (EFV)-based antiretroviral therapy (solid line) and with dolutegravir (DTG)-based antiretroviral (dashed line) in 9 pregnant women who were not on daily isoniazid prophylaxis (graph plots on the left), and in 4 pregnant women who were on isoniazid prophylaxis (graph plots on the right). Data are presented on a semi-logarithmic plot as medians (interquartile ranges [IQR]).

Table S1. Piperaquine exposure when administered with dolutegravir- compared with efavirenz-based antiretroviral therapy(DP), stratified by concomitant intake of isoniazid prophylaxis (N=13)

	**Participants not on isoniazid prophylaxis (n=9)**				**Participants on isoniazid prophylaxis (n=4)**			
	**Geometric Mean (90% CI)**		**GM Ratio (90% CI)**		**Geometric Mean (90% CI)**		**GM Ratio (90% CI)**	
**Pharmacokinetic parameter**	**Piperaquine on DTG-based ART (Sequence 3)**	**Piperaquine on EFV-based ART (Sequence 1)**	**Sequence 3 / Sequence 1**	**P-value***	**Piperaquine on DTG-based ART (Sequence 3)**	**Piperaquine on EFV-based ART (Sequence 1)**	**Sequence 3 / Sequence 1**	**P-value***
$AUC_{0\text{-}672\ hours}$ (ng.hr/mL)	25,240 (20,445-31,160)	15,415 (13, 375-17,766)	**1.64 (1.26-2.12)**	**0.005**	27,463 (23,562-32,011)	19,201 (13,145-28,047)	1.43 (0.94-2.17)	0.069
C_{max} (ng/mL)	403 (293-556)	393 (319-486)	1.03 (0.69-1.52)	0.838	476 (389-581)	382 (195-750)	1.24 (0.61-2.53)	0.557
$C_{day\ 7}$ (ng/mL)	30 (24-38)	18 (16-21)	**1.66 (1.26-2.16)**	**0.001**	33 (28-40)	20 (11-39)	1.65 (0.84-3.28)	0.143
$C_{day\ 28}$ (ng/mL)	7.4 (6.0-9.1)	2.2 (1.9-2.6)	**3.36 (2.59-4.43)**	**<0.001**	9.7 (5.5-17.2)	4.2 (3.0-5.8)	2.31 (1.15-4.79)	0.059
T_{max} (hr)	3.7 (2.9-4.7)	3.3 (2.6-4.2)	1.12 (0.80-1.56)	0.529	3.1 (1.9-5.0)	5.3 (2.8-10.0)	0.59 (0.26-1.36)	0.391
$t_{1/2}$ (hr)	234 (210-261)	174 (154-196)	**1.35 (1.14-1.59)**	**0.022**	328 (194-553)	234 (187-294)	1.40 (0.75-2.60)	0.336
CL/F (litres/hr)	142 (116-174)	226 (199-257)	**0.63 (0.49-0.80)**	**0.005**	130 (108-157)	186 (135-257)	0.70 (0.48-1.03)	0.069

GM: Geometric Mean, DTG: Dolutegravir, EFV: Efavirenz, ART: Antiretroviral therapy, CI: Confidence interval

Bold represents statistical significance

* Paired t-test

29

Table S2. Mixed-effect regression analysis assessing the impact of independent variables on piperaquine exposure

PK parameter of exposure*	Variable	Unadjusted			Adjusted		
		Coefficient (90% CI)	Standard error	P-value	Coefficient (90% CI)	Standard error	P-value
$AUC_{0\text{-}672\ hours}$	DTG- vs EFV-based ART	0.23 (0.15, 0.31)	0.05	**<0.001**	0.23 (0.15, 0.31)	0.05	**<0.001**
	Trimester change	0.06 (-0.19, 0.31)	0.15	0.679	0.11 (-0.14, 0.36)	0.15	0.459
	Isoniazid use	0.15 (-0.11, 0.42)	0.16	0.349	0.19 (-0.08, 0.45)	0.16	0.254
C_{max}	DTG- vs EFV-based ART	0.04 (-0.06, 0.14	0.06	0.498	0.04 (-0.06, 0.14)	0.06	0.498
	Trimester	0.20 (-0.18, 0.59	0.23	0.381	0.24 (-0.17, 0.65)	0.25	0.337
	Isoniazid use	0.07 (-0.36, 0.49)	0.25	0.792	0.14 (-0.30, 0.59)	0.27	0.601
$C_{day\ 7}$	DTG- vs EFV-based ART	0.25 (0.17, 0.33)	0.05	**<0.001**	0.25 (0.17, 0.33)	0.05	**<0.001**
	Trimester change	-0.06 (-0.37, 0.24)	0.19	0.728	-0.05 (-0.38, 0.29)	0.20	0.819
	Isoniazid use	0.08 (-0.25, 0.41)	0.20	0.677	0.07 (-0.29, 0.43)	0.22	0.751
$C_{day\ 28}$	DTG- vs EFV-based ART	0.09 (-0.15, 0.32)	0.14	0.538	0.09 (-0.14, 0.32)	0.14	0.535
	Trimester change#	-0.09 (-0.56, 0.38)	0.28	0.752	0.03 (-0.45, 0.52)	0.29	0.909
	Isoniazid use	0.46 (-0.02, 0.95)	0.29	0.115	0.47 (-0.05, 0.99)	0.32	0.136

* Log-transformed pharmacokinetic (PK) parameter of exposure

Bold represents statistical significance

CI= Confidence interval

Trimester change refers to switching from second to third trimester of pregnancy between treatment and PK sampling periods; i.e from sequence 1 sequence 3, respectively

Table S3. Relationship between treatment-emergent adverse events and coadministration of dihydroartemisinin with antiretroviral therapy, stratified by severity and period of occurrence during follow-up (N=13)

	Sequence 1<br><br>(2-week period following coadministration of efavirenz-based ART & DP)				Intersection between Sequences 1 & 2<br><br>(4-week lead in period while on dolutegravir-based ART following a switch from efavirenz-based ART. Period also reflecting elimation phase of piperaquine following initial DP dose)				Sequence 3<br><br>(Within the first 28 days of DP and Dolutegravir-based ART coadministration + any events presenting as unscheduled visits prior to delivery)				Delivery<br><br>(Occurring at delivery, >4 weeks after last dose of DP, while on dolutegravir-based ART)				
Relationship with coadministration of DP and antiretroviral therapy	Mild	Moderate	Severe	Life threatening	Mild	Moderate	Severe	Life threatening	Mild	Moderate	Severe	Life threatening	Mild	Moderate	Severe	Life threatening	TOTAL
**Definitely related**																	
Nausea	1																1
**Probably related**																	0
Nausea	4								3								7
Vomiting	5								3								8
Dizziness	1								1								2
Rash pruritic	1																1
**Possibly related**																	0
Nausea	1				1												2
Vomiting	2																2
Diarrhoea	2																2
Abdominal pain	1																1
Dizziness	2																2
Palpitations	1								1								2
Musculoskeletal pain	1																1
Rash pruritic									1								1
**Unlikely related**																	0
Arthralgia	1																1
Hypoesthesia	1								1								2
Dizziness	1								1								2
Vomiting					1				1								2
Toothache					1				1								2
Abdominal discomfort					1												1
Gastroenteritis					1												1
Rash pruritic					1				2								3
Headache					1				2								3
Upper gastrointestinal haemorrhage						1											1
Urinary tract infection					1	1			2								4
Night sweats									1								1
Pain in extremity									1								1
Upper respiratory tract infection									2								2
Musculoskeletal pain									1								1
Orthostatic hypotension									1								1

Hypersensitivity				1					1
Neonatal asphyxia								1	1
Preterm labour						1			1
Premature rupture of membranes							1		1
**Definitely unrelated**									0
Dizziness	1								1
Upper respiratory tract infection	1								1
Urinary tract infection	1	2							3
Vaginal discharge	1								1
Catheter site pain	2				1				3
Rhinitis	1	1							2
Rash pruritic			1						1
Toothache		1							1
Nausea			1						1
Headache		1							1
Pruritus		1							1
Hypoesthesia		1							1
Trichomoniasis		1							1
TOTAL	32	16	4	26	1	1	1	1	82

ART= Antiretroviral therapy

DP= Dihydroartemisinin-piperaquine

Table S4. Overview of published data on the pharmacokinetics of piperaquine in pregnancy, postpartum period, and non-pregnant women

Study	Number of participants	Period at time of PK sampling	*Other concomitant treatment	Pharmacokinetic parameter		
				AUC ng.h/mL	C_{max} ng/mL	$C_{day\ 7}$ ng/mL
Banda CG *et al*[a] (present study)	13	2nd-3rd trimester	Efavirenz (Sequence 1)	16,492 (14,317-18,998)[#]	390 (314-484)	19 (16-23)
	13	2nd-3rd trimester	Dolutegravir (Sequence 3)	25,904 (22,322-30,061)[#]	424 (339-532)	31 (27-37)
Kajubi R *et al*[b]	26	3rd trimester	Efavirenz	6,600 (5,570-7,830)[$]	342 (285-411)	15.1 (13.0-17.6)
	30	3rd trimester	None (Not living with HIV)	10,600 (88,400-12,700)[$]	391 (323-474)	30.5 (25.9-36.0)
	30	Postpartum (38 weeks postpartum)	None (Not living with HIV)	17,600 (15,100-20,700)[$]	499 (393-633)	39.0 (32.3-47.2)
Rijken MJ *et al*[c]	24	2nd-3rd trimester	None (Not living with HIV)	28,100 (10,800–40,200)[*#]	309 (138–575)	31.8 (13.3–80.2)
	23	Nonpregnant women	None (Not living with HIV)	21,300 (2,370–49,400)[*#]	245 (53.4–798)	25.9 (6.80–56.6)
Adam I *et al*[c]	12	2nd-3rd trimester	None (Not living with HIV)	32,400 (22,700–56,000)[*#]	374 (225–807)	50.5 (24.4–106)
	12	Nonpregnant women	None (Not living with HIV)	37,500 (13300–91,600)[*#]	312 (48.9–976)	56.9 (20.8–168)

[a] Geometric mean (90% confidence interval)

[b] Geometric mean (95% confidence interval)

[c] Median (range)

DP = dihydroartemisinin piperaquine

* Other concomitant medication (other than standard co-medication in people living with HIV which includes cotrimoxazole prophylaxis and, in some, isoniazid prophylaxis)

[#] $AUC_{0\text{-}672\ hours\ or\ day\ 28}$

[$] $AUC_{0\text{-}504\ hours\ or\ day\ 21}$

[*#] $AUC_{0\text{-}last}$

Table S5 Piperaquine exposure in the first 14 days and over 28 days during coadministration with dolutegravir (DTG) - compared with efavirenz (EFV) - based antiretroviral therapy (ART) [N=13]

Pharmacokinetic parameter	GM (90% CI)		GM Ratio (90% CI)	
	Piperaquine on DTG- based ART (Sequence 3)	Piperaquine on EFV- based ART (Sequence 1)	Sequence 3 / Sequence 1	P-value*
$AUC_{0\text{-}672\ hours}$ (ng.hr/mL)	25,904 (22,322-30,061)	16,492 (14,317-18,998)	**1.57 (1.28-1.93)**	**0.001**
$AUC_{0\text{-}336\ hours}$ (ng.hr/mL)*	21,339 (18,153-25,084)	14,744 (12,706-17,109)	**1.45 (1.16-1.81)**	**0.003**
$t_{1/2}$ (hr)	260 (219-309)	190 (169-215)	**1.37 (1.10-1.69)**	**0.014**
$t_{1/2}$ (hr)*	107 (100-116)	87 (76-101)	**1.23 (1.05-1.45)**	**0.036**
CL/F (litres/hr)	138 (119-160)	213 (188-241)	**0.65 (0.53-0.79)**	**0.001**
CL/F (litres/hr)*	168 (143-197)	238 (211-270)	**0.70 (0.57-0.86)**	**0.003**

*PK parameters based on piperaquine concentrations from 0 to 336 hours (day 14)

GM: Geometric Mean, DTG: Dolutegravir, EFV: Efavirenz, ART: Antiretroviral therapy, CI: Confidence interval

Bold represents statistical significance

* Paired t-test
